# Analgesic drug use in elderly persons: A population-based study in Southern Italy

**DOI:** 10.1371/journal.pone.0222836

**Published:** 2019-09-19

**Authors:** Ylenia Ingrasciotta, Janet Sultana, Francesco Giorgianni, Enrica Menditto, Angelo Scuteri, Michele Tari, Daniele Ugo Tari, Giorgio Basile, Gianluca Trifiro’

**Affiliations:** 1 Department of Biomedical and Dental Sciences and Morphofunctional Imaging, University of Messina, Messina, Italy; 2 Unit of Clinical Pharmacology A.O.U. ‘G. Martino’ Hospital’, Messina, Italy; 3 CIRFF, Center of Pharmacoeconomics, University of Naples Federico II, Naples, Italy; 4 HSR Pisana IRCCS, Rome, Italy; 5 Local Health Unit of Caserta, Caserta, Italy; 6 Department of Medical Informatics, Erasmus Medical Center, Rotterdam, the Netherlands; University of Catanzaro, ITALY

## Abstract

**Introduction:**

Analgesics such as non-steroidal anti-inflammatory drugs (NSAIDs), weak and strong opioids are commonly used among elderly persons. The aim of this study was to describe the demographic and clinical characteristics of elderly analgesic users and to measure the frequency of analgesic use, including the frequency of potentially inappropriate analgesic use.

**Methods:**

The Arianna database was used to carry out this study. This database contains prescription data with associated indication of use for 1,076,486 inhabitants registered with their general practitioners (GPs) in the Caserta Local Health Unit (Caserta district, Campania region in Italy). A cohort of persons aged ≥65 years old with >1 year of database history having at least one analgesic drug (NSAIDs, strong or weak opioids) between 2010 and 2014 were identified. The date of the first analgesic prescription in the study period was considered the index date (ID).

**Results:**

From a source population of 1,076,486 persons, 116,486 elderly persons were identified. Of these, 94,820 elderly persons received at least one analgesic drug: 36.6% were incident NSAID users (N = 36,629), while 13.2% were incident weak opioid users (N = 12,485) and 8.1% were incident strong opioid users (N = 7,658). In terms of inappropriate analgesic use, 9.2% (N = 10,763) of all elderly users were prescribed ketorolac/indomethacin inappropriately, since these drugs should not be prescribed to elderly persons. Furthermore, at least half all elderly persons with chronic kidney disease or congestive heart failure were prescribed NSAIDs, while these drugs should be avoided.

**Conclusion:**

Analgesics are commonly used inappropriately among elderly persons, suggesting that prescribing practice in the catchment area may yet be improved.

## 1. Introduction

Pain is common medical problem among older persons and can lead to impaired functionality, depression and a lower quality of life [1]. Mild to moderate acute pain is treated with acetaminophen and non-steroidal anti-inflammatory drugs (NSAIDs) as first-line agents [2]. NSAIDs are generally categorized as: a) non-selective compounds which inhibit both cyclo-oxygenase (COX)-1 and COX-2 enzymes; b) COX-2-selective drugs, also known as coxibs, which are associated with a lower risk of gastrointestinal bleeding than non-specific NSAIDs [3]. Due to their strong anti-inflammatory action, NSAIDs are generally indicated in pain of inflammatory origin. On the other hand, opioid analgesics are indicated in pain of visceral origin, in palliative care and in general, in moderate to severe pain not responding to NSAIDs.

Drug use and safety among elderly persons is of importance because this population is more likely to use several drugs concomitantly [4]. Elderly persons are also likely to be frailer in terms of increased multi-morbidity, impaired cognition and reduced independence in activities of daily living [5]. Indeed, the high prevalence of pain in frail elderly persons [6] in addition to the widespread overuse of opioids in some countries [7] creates an urgent need to understand pharmacological pain management approach among the elderly. This is important given the drug risk-benefit profiles may change as a function of cognitive and functional impairment [8].

The Beers criteria for inappropriate analgesic prescribing suggest that the NSAIDs ketorolac and indomethacin should not be prescribed in elderly persons and that non-selective NSAIDs should not be used chronically in elderly persons [9]. Furthermore, several analgesics are contraindicated in conditions which are more frequently present in elderly persons compared to younger ones, such as congestive heart failure (CHF) and chronic kidney disease (CKD). It is therefore important to describe whether analgesic drugs are used appropriately among elderly persons, especially in view of the potential risks in this population, such as falls/fractures with opioid use [10], and gastric bleeding [11], cardiovascular events [12] or acute kidney disease/CKD [13, 14] with NSAIDs.

Despite the increasing prevalence of pain with increasing age, two leading European clinical guideline organizations, the UK National Institute for Health and Care Excellence (NICE) and the Scottish Intercollegiate Guidelines Network (SIGN) do not have guidelines dedicated to the management of pain in the elderly. The Italian Geriatric Society (SIGOT) does not have such guidelines on pain management in the elderly while other societies, such as the British Geriatrics Society does [15]. Analgesic drug utilization in this population may therefore be variable. This in addition to the widespread opioid epidemic in some countries is a further incentive to study analgesic use among elderly persons. The appropriateness of analgesic use in Italy has been the topic of limited published research, including the appropriateness of opioid use in cancer patients [16], inappropriate use in chronic pain [17] or in relation to a change in drug prescribing directives [18, 19]. However, to our knowledge, there is no recent Italian study investigating inappropriate analgesic use in the elderly.

The aim of this study was therefore to describe the demographic and clinical characteristics of elderly analgesic users and to measure the frequency of analgesic use in this population, including the frequency of potentially inappropriate analgesic use.

## 2. Methods

### 2.1. Data source

The Arianna database was used to carry out this study. This database contains prescription data with associated indication of use for 1,076,486 persons living in the catchment area registered with their GPs in the Caserta Local Health Unit (Caserta district, Campania region in Italy). These data are linked with the following patient-level claims data from the same catchment area: demographic registry, pharmacy claims database for drugs acquired through the Italian National Healthcare System (NHS) and a database of hospital discharge diagnoses. Within the linkage database, diagnoses are recorded using the 9^th^ Edition of the International Classification of Disease codes with clinical modification (ICD-9 CM) while drugs are recorded using Anatomical Therapeutic Chemical (ATC) codes. Pharmacy claims contain prescription data for drugs that are covered by the Italian NHS, including most analgesics. Acetaminophen is not covered by the Italian NHS unless it is found in combination with other drugs. Although most analgesics are covered by the Italian NHS, patients may still opt to buy them out-of-pocket.

In addition to the demographic and clinical patient characteristics mentioned above, the results of a comprehensive geriatric assessment (CGA) concerning cognitive status, mobility, nursing needs and social support were used to further describe the study population. CGA data was extracted for approximately 75% of persons aged 65 and older (N = 116,486 in the study period) registered with the Caserta Local Health Unit. This CGA is carried out yearly for elderly persons in the catchment area by their GPs [20].

The study was carried out using retrospectively collected and anonymized data. In Italy, such studies do not require ethical approval by an Ethics Committee as per the Italian Health Ministry/Italian Drug Agency decree of the 3rd August 2007.

### 2.2. Study population

A cohort of patients from the Caserta catchment area was identified, including patients who had one year of database history, were aged at least 65 years old and received at least one analgesic drug prescription between 2010 and 2014. Patients were censored if transferred out of the database (i.e., changed to a permanent residence outside the catchment area) or if they died. Persons with no analgesic drug dispensing within one year before the index date were considered incident drug users.

### 2.3. Exposure

Analgesic drugs, i.e. NSAIDs, weak opioids and strong opioids were the exposure of interest and were identified within the population of elderly persons using ATC codes. The date of the first analgesic prescription in the study period was considered the index date (ID). Acetaminophen was not included as a main study drug as, this drug is not covered by the Italian NHS unless in combination with codeine and is mainly purchased out-of-pocket as an over-the-counter (OTC) drug. Codeine was considered only in combination with acetaminophen as only this preparation is indicated for pain in Italy.

All analgesic drugs were grouped by pharmacological categories: NSAIDs, including non-selective NSAIDs and coxibs, weak opioids or strong opioids (see **S1 Table** for further detail). Codeine was considered only in combination with acetaminophen as only this preparation is indicated for pain in Italy. Analgesics were further categorized by formulation (oral, injection, transdermal, rectal or nasal). Indications associated with the analgesic drug prescriptions were reported. The mean prescribed defined daily dose (PDD) for each analgesic prescription was estimated by dividing the drug doses prescribed (i.e. number of units per day multiplied by the strength prescribed) by the defined daily dose (DDD).

Inappropriate analgesic drug prescribing was identified using Beers criteria [8]. The frequency of inappropriate drug prescriptions in the elderly population was estimated based on the following recommendations: 1) Completely avoid indomethacin and ketorolac in older persons due to an increased risk of GI bleeding and peptic ulcer disease; 2) Avoid chronic use (defined within this study as >90 days) of oral non- selective NSAIDs, i.e. aspirin at doses exceeding 325 mg daily, diclofenac, ibuprofen, ketoprofen, meloxicam, nabumetone, naproxen, oxaprozin or piroxicam, in high risk groups, such as those aged >75 or taking oral or parenteral corticosteroids, anticoagulants, or antiplatelet agents. Inappropriate drug use was also identified based on contraindications in specific disease states: 1) NSAID use in chronic kidney disease (CKD) of any stage, non-selective NSAIDs and coxib use in persons with heart failure; 2) Aspirin at doses exceeding 325 mg daily and non-selective NSAID use in persons with gastric/duodenal ulcers; 3) any pentazocine prescriptions to elderly persons.

### 2.4. Data analysis

For incident analgesic users, demographic and clinical characteristics in terms of age, sex, co-morbidities, specifically heart failure, diabetes mellitus, ischemic heart disease (e.g. angina pectoris and acute myocardial infarction), cerebrovascular events (e.g. transient ischemic attack and stroke), chronic kidney disease, gastric and duodenal ulcer, liver disease and gout were identified any time prior to the index date. The number of concomitant drugs used was also estimated within three months before the index date as a proxy of overall disease burden for the following: beta-blockers, diuretics, angiotensin-converting enzyme (ACE) Inhibitors, angiotensin receptor blockers, proton pump inhibitors, misoprostol, statins, lithium, digoxin, methotrexate, gabapentin/pregabalin, tricyclic antidepressants, antipsychotics, selective serotonin reuptake inhibitors (SSRI), anticoagulants, antiplatelet drugs and corticosteroids.

The use of analgesics among incident users was described in terms of indication of use median number of daily doses (with interquartile range), formulation used and median number of inappropriate prescriptions (with interquartile range). Incident analgesic users were also described in terms of selected CGA evaluations. Only CGA data regarding elderly incident analgesic users was extracted; results were restricted to the CGAs closest to the index date.

The frequency of inappropriate analgesic use was measured without restricting to incident analgesic users since the inappropriate use of these drugs concerns a broader group of patients, i.e. those who are incident as well as those who are not new users. All frequencies related to inappropriate drug use were stratified by age groups: 65–74 years old, 75–84 years old and ≥85 years. Frequencies were calculated both considering number of persons with the disease as a denominator as well as number of elderly persons using the analgesic drugs of interest.

A time to event Kaplan-Meier analysis (i.e., time to discontinuation) was performed, stratifying analgesic-naïve users by pharmacological categories, to assess treatment persistence over time. From the beginning of the therapy, for each naïve user we estimated the number of days of continuous analgesic treatment, taking into account dispensed amount of active principle and Defined Daily Dose (DDD) of analgesics. Persistence to analgesics therapy was assessed based on the maximum allowed treatment gap of 60 days, defined as the time between the last day covered by analgesic drug treatment and the time to the next refill. Follow-up of naïve analgesic users was censored if patients were still on therapy at the end of the study, in case of death or no availability of further data, whichever came first.

The Kaplan Meier analysis was carried out and results were stratified by pharmacological categories (non-steroidal anti-inflammatory drugs, weak opioids and strong opioids).

### 2.5. Sub-analyses

A sub-analysis was carried using a different data source out to find to what extent non-opioid analgesics were purchased over the counter (OTC) from community pharmacies in the catchment area. This analysis was carried out using a database provided by IMS Health on pharmacy sales data for all pharmacies in Caserta. Prescription data from IMS is aggregate prescription-level data through which it is possible to distinguish between units of drugs dispensed through the NHS and those OTC.

Data management and analyses were carried out using SAS version 9.2 and SPSS/PC, Version 21 (SPSS Inc., Chicago, Illinois, USA). A p-value of 0.05 was used to denote statistical significance, using the Kruskal-Wallis, Fischer and Chi-square tests, as appropriate.

## 3. Results

### 3.1. Prevalence of analgesic use

**Fig 1:**
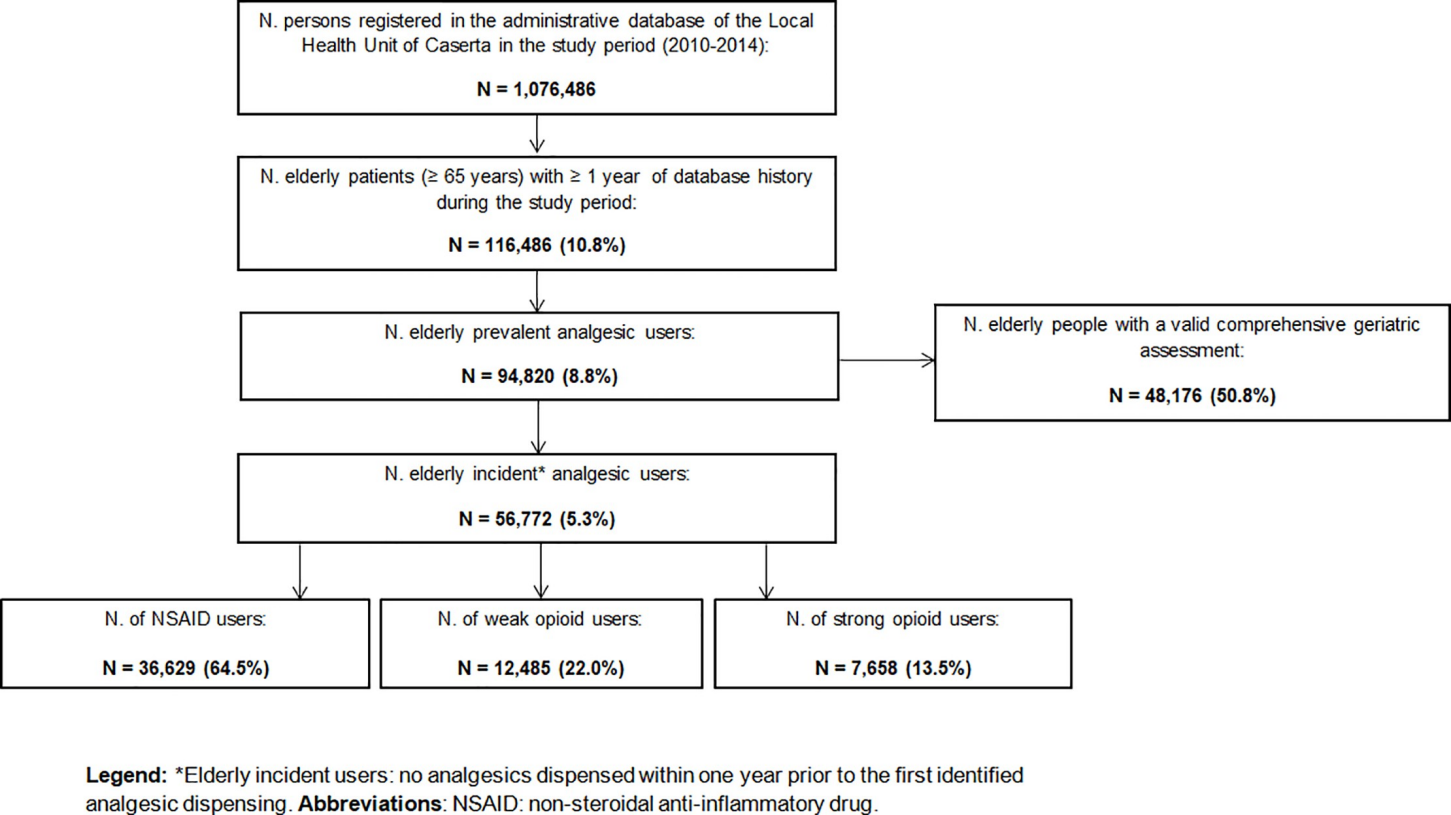
Identification of elderly persons receiving analgesic drugs during the study period. *Elderly incident users: no analgesics dispensed within one year prior to the first identified analgesic dispensing.

From a source population of 1,076,486 persons in the catchment area, 116,486 elderly persons were identified. Of these, 94,820 were elderly persons who were dispensed at least one analgesic drug (**Fig 1**). In 2014, it was seen that NSAIDs were by far the most commonly used analgesics in all age categories (**S1 Fig**). Up to 50% of all elderly persons aged 75 and over were prescribed an NSAID. The use of all other analgesics was much less common, at less than 15% for all persons aged 65 and over. There was no clear unifying trend concerning the yearly prevalence of non-opioid drugs from 2010 to 2014, although several marked changes in use can be seen for single drugs (**S2 Fig**). At the beginning of the study period, 2010, the most commonly used non-opioid drug was nimesulide, with a prevalence of approximately 20%, but this decreased by half by 2014. In 2014, the most commonly used non-opioid drugs were ketoprofen (17%), followed by diclofenac (13%) and nimesulide (11%). While the prevalence of several non-opioid drugs did not change notably or decreased during the study period, etoricoxib was the only non-opioid drug whose use increased over the study period, going from 7% to 8%. The most commonly used opioid drug was codeine in combination with acetaminophen, which increased in prevalence from 4 to 5.5% (**S3 Fig**). The use of oxycodone in combination with naloxone/acetaminophen, and tapentadol increased very markedly over the study period, going from 1 to 3.5% and 0.2 to 1.5%, respectively.

### 3.2. Incidence of analgesic use: Population characteristics

Overall, 94,820 (81.4% of total elderly) elderly analgesic users were identified, of whom 36,629 (36.6%; mean age 73.1±7.1), 12,485 (13.2%; mean age 74.4±7.0) and 7,658 (8.1%; mean age 74.2±6.8) were incident users of NSAIDs, weak and strong opioids respectively (**Table 1**). More of the incident elderly analgesic users were female rather than male, with the difference being increasingly pronounced in the following order: strong opioids > weak opioids > NSAIDs. The DDD for these three analgesic groups did not decrease linearly, but was highest for non-opioid analgesics > strong opioids > weak opioids. In terms of overall medical condition, defined using number of concomitant medications as a proxy of disease burden, strong opioid users received more concomitant drugs (7.1±4.1) than weak opioid users (6.5±4.0) or non-opioid users (4.2±3.3). Among all three analgesic groups, the most common indication for analgesic prescribing was bone and joint disorders.

**Table 1:** Characteristics of elderly incident users (≥ 65 years) of NSAIDs, weak and strong opioids in the years 2010–2014. Numbers in brackets refer to the percentages unless otherwise specified.

	NSAIDs N = 36,629 (%)		Weak Opioids N = 12,485 (%)	Strong Opioids N = 7,658 (%)	p-value
**Demographic characteristics**					
**Sex**					
Male	16,612 (45.4)		4,439 (35.6)	2,592 (33.8)	<0.001
Female	20,017 (54.6)		8,046 (64.4)	5,066 (66.2)	
**Median age, years (Q1-Q3)**	72 (67–78)		74 (68–79)	74 (69–79)	<0.001
65–74	22,836 (62.3)		6,751 (54.1)	4,184 (54.6)	<0.001
75–84	10,900 (29.8)		4,629 (37.1)	2,866 (37.4)	
≥ 85	2,893 (7.9)		1,105 (8.9)	608 (7.9)	
**Median follow-up, years (Q1-Q3)**	2.8 (2.2–4.5)		2.8 (2.3–4.9)	2.8 (2.3–4.8)	<0.001
**Comorbidities**					
*Cardiovascular diseases*					
Heart failure	2,184 (6.0)		1,055 (8.5)	651 (8.5)	<0.001
Ischemic heart disease	7,319 (20.0)		3,260 (26.1)	2,005 (26.2)	<0.001
Cerebrovascular events	32,031 (87.4)		11,703 (93.7)	7,163 (93.5)	<0.001
Hypertension	29,875 (81.6)		11,172 (89.5)	6,858 (89.6)	<0.001
*Metabolic diseases*					
Diabetes mellitus	9,268 (25.3)		3,844 (30.8)	2,370 (30.9)	<0.001
*Other chronic diseases*					
Chronic kidney disease	1,016 (2.8)		554 (4.4)	315 (4.1)	<0.001
*Miscellaneous*					
Gastric and duodenal ulcer	509 (1.4)		235 (1.9)	159 (2.1)	<0.001
Liver disease	2,762 (7.5)		1,244 (10.0)	815 (10.6)	<0.001
Gout	5,075 (13.9)		2,702 (21.6)	1,690 (22.1)	<0.001
Prior fractures	3,027 (8.3)		1,360 (10.9)	916 (12.0)	<0.001
**Previous use of pain relief medications**					
NSAIDs	-		5,388 (43.2)	3,973 (51.9)	<0.001
Weak Opioids	950 (2.6)		-	1,378 (18.0)	<0.001
Strong Opioids	404 (1.1)		586 (4.7)	-	<0.001
**Median number of concomitant drugs (Q1-Q3)**	4 (2–6)		6 (4–9)	7 (4–9)	<0.001
0	3,826 (10.4)		353 (2.8)	145 (1.9)	<0.001
1–2	9,063 (24.7)		1,473 (11.8)	708 (9.2)	
3–5	12,875 (35.1)		3,865 (31)	2,064 (27.0)	
6–10	9,156 (25.0)		5,005 (40.1)	3,274 (42.8)	
>10	1,709 (4.7)		1,789 (14.3)	1,467 (19.2)	
**Concomitant drugs**					
*Cardiovascular drugs*					
Anti-hypertensive drugs	23,419 (63.9)		9,140 (73.2)	5,608 (73.2)	<0.001
Statins	8,715 (23.8)		3,292 (26.4)	2,007 (26.2)	<0.001
Digoxin	1,110 (3.0)		549 (4.4)	319 (4.2)	<0.001
*Central nervous system drugs*					
Antidepressant drugs	4,393 (12.0)		2,141 (17.1)	1435 (18.7)	<0.001
Antipsychotics	47 (0.1)		16 (0.1)	14 (0.2)	0.127
Gabapentin/pregabalin	471 (1.3)		407 (3.3)	402 (5.2)	<0.001
*Rheumatological drugs*					
Methotrexate	83 (0.2)		56 (0.4)	51 (0.7)	<0.001
Corticosteroids	2,273 (6.2)		1,687 (13.5)	1,408 (18.4)	<0.001
*Blood thinning drugs*					
Anticoagulants	2,784 (7.6)		1,669 (13.4)	1,128 (14.7)	<0.001
Antiplatelet drugs	10,966 (29.9)		4,595 (36.8)	2,612 (34.1)	<0.001
Anticoagulants/antiplatelet drug combination therapy	440 (1.2)		185 (1.5)	123 (1.6)	0.004
*Miscellaneous*					
Gastroprotectant agents	11,758 (32.1)		6,166 (49.4)	4,320 (56.4)	<0.001
**Analgesic use**					
Median number of inappropriate analgesic prescriptions (Q1-Q3)*		8 (5–12)	11 (7–15)	11 (8–16)	<0.001
Median number of daily doses, DDD (Q1-Q3)		0.03 (0.02–0.06)	0.01 (0.00–0.01)	0.01 (00.0–0.02)	<0.001
Median duration of treatment, days (Q1-Q3)		17 days (16–31)	5 days (5–11)	8 days (5–22)	
**Formulation**					
Oral		27,477 (75.0)	11,826 (94.7)	6,699 (87.5)	<0.001
Injection		8,783 (24.0)	610 (4.9)	12 (0.2)	
Transdermal		-	-	849 (11.1)	
Rectal		6 (0.0)	-	-	
Nasal		-	-	13 (0.2)	
More than one formulation		363 (1.0)	47 (0.4)	85 (1.1)	
**Indication of use**					
Bone and joint diseases	33,230 (90.7)		10,149 (81.3)	5,678 (74.1)	<0.001
Urinary tract disorders (i.e. renal colic)	833 (2.3)		78 (0.6)	19 (0.3)	
Cancer pain	392 (1.1)		952 (7.6)	1,154 (15.1)	
Other and unclassified	2,162 (5.9)		1,305 (10.5)	807 (10.5)	

*inappropriate drug prescriptions were defined as: 1) use of indomethacin and ketorolac among elderly persons; 2) chronic use of oral non- selective NSAIDs (aspirin at doses exceeding 325 mg daily, diclofenac, ibuprofen, ketoprofen, meloxicam, nabumetone, naproxen, oxaprozin or piroxicam, among patients aged >75 or those taking oral or parenteral corticosteroids, anticoagulants, or antiplatelet agents; 3) any NSAID use in chronic kidney disease; 4) non-selective NSAID and coxib use in patients with heart failure; 5) aspirin at doses exceeding 325 mg daily and non-selective NSAID use in persons with gastric/duodenal ulcers; 6) any pentazocine prescriptions to elderly persons. **Abbreviations:** NSAID: non-steroidal anti-inflammatory drugs; Q1-Q3: 25^th^ percentile to 75^th^ percentile

### 3.3. Incidence of analgesic use: drug utilization in frail elderly persons

Overall, strong opioid users showed more factors indicating frailty status than weak opioid or non-opioid users. For example, a larger proportion of elderly strong opioid users had mild cognitive impairment compared to weak opioids and non-opioid drug users, while there was no difference in the proportion of persons with moderate and severe cognitive impairment among the three analgesic groups. With regard to nursing needs, non-opioid users were more commonly those with no additional nursing needs, compared to the other two analgesic groups; conversely, strong opioid users more commonly required nursing assistance than the other two analgesic groups. Strong opioid users were also more likely to be required assistance regarding mobility **(Table 2)**.

**Table 2:** Comprehensive geriatric assessment for elderly incident analgesic users.

	NSAID users N = 11,409 (%)	Weak opioid users N = 4,033 (%)	Strong opioids users N = 2,528 (%)	p-value
**Cognitive status: SPMSQ results**				
Intact intellectual functioning	9,152 (80.3)	3,062 (75.9)	1,915 (75.8)	<0.001
Mild intellectual impairment	1,340 (11.7)	604 (15.0)	380 (15.0)	
Moderate intellectual Impairment	573 (5.0)	249 (6.2)	155 (6.1)	
Severe intellectual impairment	344 (3.0)	118 (2.9)	78 (3.1)	
**Nursing needs**				
High	537 (4.7)	270 (6.7)	197 (7.8)	<0.001
Low	1,345 (11.8)	678 (16.8)	488 (19.3)	
No nursing needs	9,527 (83.5)	3,085 (76.5)	1,843 (72.9)	
**Social support**				
Good	11,015 (96.5)	3,840 (95.2)	2,431 (96.2)	<0.001
Poor	394 (3.5)	193 (4.8)	97 (3.8)	
**Mobility**				
Independent	9,953 (87.2)	3,301 (81.8)	1,972 (78.0)	<0.001
Requires assistance	688 (6.1)	350 (8.7)	274 (10.8)	
No mobility at all	768 (6.7)	382 (9.5)	282 (11.2)	

**Abbreviations:** SPMSQ: short portable mental status questionnaire.

### 3.4. Inappropriate analgesic use

Overall, a total of 10,763 (9.2%) of all elderly analgesic users were considered to have an inappropriate prescription for the NSAIDs (ketorolac or indomethacin), although this appeared to be more widespread for ketorolac (9,748 patients, 8.4%) compared to indomethacin (1,237 patients, 8.4%) (**Table 3**). In contrast, the chronic use of non-selective non-steroidal anti-inflammatory drugs, defined as that exceeding 90 days, was less common (1,1611 patients, 1.4%). There were only 4 elderly persons with a prescription for pentazocine. With regards to disease-specific indicators of prescribing appropriateness, the degree of inappropriate prescribing was similar for NSAIDs use in CKD, non-selective NSAID or coxib use in heart failure and non-selective NSAID use in gastric/duodenal ulcers (**Table 4**).

**Table 3:** Frequency of inappropriate drug prescriptions in the elderly population according to the Beers criteria, stratified by age groups.

		Patients 65–74 years old N = 70,406		Patients 75–84 years old N = 34,612		Patients ≥85 years old N = 11,468		Total N = 116,486	
Drugs/drug classes	Inappropriate drug use criteria	Patients N (%)	Prescriptions N	Patients N (%)	Prescriptions N	Patients N (%)	Prescriptions N	Patients N (%)	Prescriptions N
Indomethacin or ketorolac	Avoid in elderly persons	6,345 (9.0)	12,375	3,553 (10.3)	7,428	865 (7.5)	1,686	**10,763 (9.2)**	**21,489**
Indomethacin		733 (1.0)	1,422	425 (1.2)	977	79 (0.7)	193	**1,237 (1.1)**	**2,592**
Ketorolac		5,745 (8.2)	10,953	3,201 (9.3)	6,451	802 (7.0)	1,493	**9,748 (8.4)**	**18,897**
All oral non-COX 2- selective NSAIDs	Avoid chronic use if no gastroprotectant agents* are used	977 (1.4)	5,728	526 (1.5)	3,573	108 (0.9)	717	**1,611 (1.4)**	**10,018**

*proton pump inhibitor or misoprostol

**Abbreviations**: COX-selective NSAIDs: cyclo-oxygenase 2 selective non-steroidal anti-inflammatory agents

**Table 4:** Frequency of inappropriate drug prescriptions in the elderly population with a specific disease, stratified by age groups.

	Patients 65–74 years old			Patients 75–84 years old			Patients ≥85 years			Total	
	Patients N = 70,406 (% on all population; % on patients with the disease)	Prescriptions N		Patients N = 34,612 (% on all population; % on patients with the disease)		Prescriptions N	Patients N = 11,468 (% on all population, % on patients with the disease)	Prescriptions N		Patients N = 116,486 (% on all population, % on patients with the disease)	Prescriptions N
**Chronic kidney disease any stage**											
Non-selective NSAIDs and COX-2 inhibitors	1,993 (2.8; 55.0)	12,173		1,966 (5.7; 51.8)		10,892	733 (6.4; 44.1)	3,277		4,692 (4.0; 51.7)	26,342
**Heart failure**											
Non-selective NSAIDs and COX-2 inhibitors	2,891 (4.1; 58.0)	18,292		3,492 (10.1; 56.3)		21,077	1,472 (12.8; 47.6)	7,052		7,855 (6.7; 55.0)	46,421
**Gastric/duodenal ulcers**											
Aspirin >325 mg/day	47 (0.1; 0.7)	77		32 (0.1; 0.8)		42	9 (0.1; 0.7)	33		88 (0.1; 0.7)	152
Non-selective NSAIDs	4,269 (6.1; 62.2)	24,832		2,562 (7.4; 62.6)		14,846	727 (6.4; 53.7)	3,910		7,558 (6.5; 61.4)	43,588

**Abbreviations**: COX-selective NSAIDs: cyclo-oxygenase 2 selective non-steroidal anti-inflammatory agents; NSAIDs: non-steroidal anti-inflammatory drugs

### 3.5. Persistence

**Fig 2:**
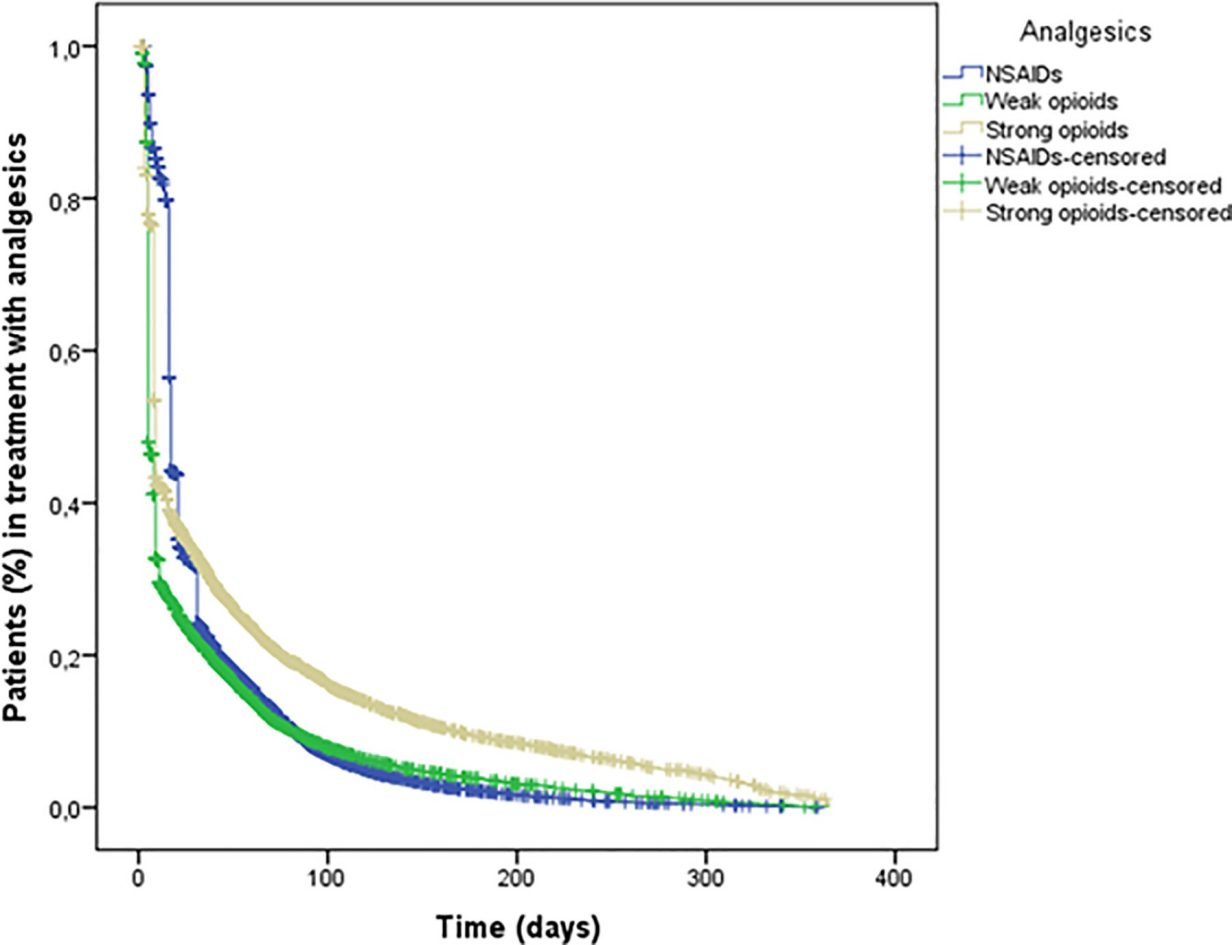
Time to discontinuation of analgesic therapy among incident analgesic users.

The median duration of treatment was 17 days (IQR: 16–31) for NSAIDs, 5 days (IQR: 5–11) for weak opioids and 8 days (IQR: 5–22) for strong opioids. Overall, 91.01% (N = 51,370) of elderly patients discontinued their analgesic medication using a 60 day treatment gap to define discontinuation; this decreased to 84.09% (N = 48,946) on using a 120 day treatment gap definition. Using any definition, persistence was always slightly higher for strong opioid use. None of the analgesic users were persistent at 1 year from the start of analgesic use (**Fig 2**). Persistence for strong opioids was always highest while that for weak opioids and NSAIDs was lower.

### 3.6. Sub-analysis

The analysis on analgesic dispensing using IMS pharmacy sales data confirmed that by and large, about half of non-opioid analgesic drugs acquired in community pharmacies was indeed bought over-the-counter and could not have been captured by the NHS administrative drug dispensing databases (**S4 Fig**).

## 4. Discussion

To our knowledge, the present study is the first to describe analgesic use and appropriateness among elderly persons in Italy. Among the incident elderly analgesic users identified, more persons were prescribed non-opioid analgesics than opioid analgesics, and among opioid analgesics, weak opioids were more commonly used than strong opioids. This is in line with the recommended stepped use of analgesic drugs, where non-opioids are first-line agents, followed by weak and strong opioids. Recent years have seen an ‘opioid crisis’ take place in the U.S.A., with widespread over-use and misuse of opioids, leading to a large number of overdose-related deaths [21]. In Italy there has been a four-fold increase in the number opioid prescriptions from 2007 to 2017, as reported by the Italian Society of Pharmacology, however this increase is modest compared to other European countries [22]. Indeed, the cautious use of opioids is confirmed by the very short median duration of these drugs: 5 days (IQR: 5–11) for weak opioids and 8 days (IQR: 5–22) for strong opioids; there is no study to which these results can be compared at the time of writing. The trend in opioid use in Italy may be related to a law passed in 2010 known as Law 38/2010 in which the Italian government commits to improving the access to palliative care and pain relief. While a recently published study using data from pharmacy sales on a national level confirmed a relative increase in opioid use in Italy from 2000 to 2010, this study reported that opioid use is overall low; the most commonly used drug was codeine, which was used at 5 DDD per day per 1,000 persons in 2010 [23].

Evaluating the appropriateness of opioid prescribing is a challenge, as this depends on an accurate classification of the severity of pain. For example, the present study found that weak and strong opioids were commonly used for bone and joint disorders, although less commonly than non-opioid analgesics. Although opioids can be used appropriately in joint and bone-related pain, they should only be used for moderate to severe pain [24]. On the other hand, opioids were commonly used in persons with cancer as an indication, in line with the indication of these drugs in palliative care [25]. In the context of frailty, it is surprising that strong opioids were used more commonly in frailer persons compared to persons with a better cognition and functional status because elderly persons who are frail are likely to have poorer mobility [26]. This is likely to predispose such elderly persons to ADRs such as falling with risk of facture, increasing the risk of hospitalization and disability [27].

The decreasing prevalence of nimesulide among elderly persons is perhaps the most notable trend among non-opioid analgesics. This may be related to concerns as early as 2007, when EMA reviewed nimesulide after the government of Ireland suspending the marketing authorization for this drug due to concerns about drug-induced liver disease [28]. Uncertainty about this drug remained unresolved, because in 2010, EMA requested the Committee for Medicinal Products for Human Use to evaluate the nimesulide risk-benefit profile and recommend a regulatory course of action such as changing, suspending or withdrawing the marketing authorization of the drug throughout the European Union. The Italian Drug Agency published a notice on the risk of hepatotoxicity with use of NSAIDs, with specific mention of nimesulide in 2012, however the reduction in the use of nimesulide after 2012 was minimal compared to the reduction from 2010 to 2012 [29].

On the other hand, the mild increase in the prevalence of etoricoxib use from 2010 to 2014 may make sense in the context of sequence of safety concerns about this drug, and indeed the whole class of COX-2 inhibitors. The controversy surrounding these drugs culminated in the withdrawal of rofecoxib; it may be hypothesized that the subsequent evaluation of the safety of etoricoxib in 2008 by EMA [30], published in Italian by the Italian Drug Agency [31], may have been an important factor leading prescribers to prescribe this drug more confidently.

In terms of absolute numbers, ketorolac and indomethacin were commonly inappropriately prescribed, that is, they were prescribed in 10,763 elderly persons whereas they should not be prescribed in this population at all. Nevertheless, when considered in terms of relative frequency, this population consisted of 9.2% of the elderly persons considered. Non-selective NSAIDs were prescribed inappropriately, that is chronically and without concomitant gastroprotective drugs, in a smaller number of elderly persons (N = 1,611, 1.4% of the study population). As expected the inappropriate use of pentazocine, defined as the prescription of this drug to an elderly person, was very low, amounting to only 4 persons during the study period. However, we suggest that this does not reflect the appropriateness of use of this drug as much as the low prevalence of this drug; the clinical relevance of this finding is limited. The appropriateness of other opioid drugs was not treated in Beers criteria, and was seldom mentioned in START-STOPP criteria [32], except with regard to the recommended concomitant use of laxatives if opioids are used chronically and concerning the treatment of pain in the appropriate clinical context, i.e. not treating mild pain with strong transdermal opioids as a first line of treatment. It was not possible to evaluate this criterion for inappropriate use since the level of pain was not quantifiable as mild, moderate or severe. Similarly, the appropriateness of other analgesic drugs in the context of pain severity was not possible. It is worth noting that the appropriate use of medications in frail persons may go beyond the available guidance on the appropriate use of medications. For example, while acute and chronic kidney disease may be caused, exacerbated or worsened by non-opioid analgesics, it may be misleading to monitor renal function renal function in elderly persons through creatinine levels alone in patients with sarcopenia, i.e. reduced muscle mass and strength. Sarcopenia is often a component of frailty especially in very old patients; in patients with this condition it is essential to use equations such as CKD EPI or MDRD to monitor renal function [33]. Results on medication appropriateness in the present study do not take this into account.

The main strength of this study is the use of real-world clinical data reflecting the actual use of analgesic drug prescribing in clinical practice and the large size of the elderly population studied. Another important strength of this study is the detailed description of elderly analgesic users in terms of frailty. This is approach combines traditional drug utilization research using healthcare databases with data from comprehensive geriatric assessments. The latter is rarely available in large-scale databases and is even more rarely used. A further strength is the detail provided not only regarding the prevalence of analgesic drug use but also regarding use of these drugs in elderly persons with varying degrees of cognitive or physical impairment, which is not commonly available or used in secondary healthcare data. Furthermore, the potentially inappropriate use of these drugs in elderly persons was described in detail, including the duration of drug use as well as the use of analgesics in specific populations such as the use of NSAIDs and COX-2 inhibitors not concomitantly prescribed gastroprotective drugs.

However this study also has some limitations. Although we assume that a prescription for analgesics covers the patient for the duration equivalent to finishing all the doses in a package, it is possible that this leads to an over-estimation of drug exposure, as analgesic use may be sporadic. Furthermore, it is possible that persons in the catchment area buy the analgesic drugs out of pocket, rather than through the Italian NHS. In this case, such drug use would not be captured. However, it is unlikely that persons chronically using these drugs would buy them out-of-pocket as over the counter drugs, particularly concerning strong NSAIDs, coxibs and opioids. Acetaminophen, along with other medications which are not covered by the Italian NHS or which are covered but which patients prefer to buy out of pocket, such as inexpensive medications, are not captured by the present data. Furthermore, the diagnoses identified in the present study may be underestimated, since these are only captured on hospital admission. As a result, inappropriate medication use may also be underestimated. Finally, it should be borne in mind that the present study is descriptive in nature and predictors of drug utilization were not explored. Future studies may want to build on findings from the present study by investigating predictors of using non-opioid and/or opioid medications as well as describe analgesic polypharmacy and its implications in elderly populations.

## 5. Conclusions

Analgesics are commonly used in elderly persons, with weak non-opioid analgesics being most used. In particular these drugs were commonly used in persons having varying degrees of cognitive and physical impairment. Overall, at least half all elderly persons with chronic kidney disease or congestive heart failure were prescribed NSAIDs inappropriately. Both non-opioid and opioid analgesics should be used with caution in elderly persons, and the need and appropriateness of such drugs should be evaluated regularly.

## Supporting information

S1 Table Analgesics identified by ATC codes and generic name. (PDF)

S1 Fig Prevalence of elderly analgesic users, stratified by age group in 2014. (TIF)

S2 Fig Prevalence of elderly NSAID users, stratified by calendar year and individual non-selective NSAIDs and coxibs. Other NSAIDs: lornoxicam, meloxicam, diclofenac and misoprostol, ketoprofen sucralfate, ketoprofen and omeprazole, dexketoprofen, naproxen, nabumetone, flurbiprofen, acetylsalicylic acid, acetylsalicylic acid combinations excl. Psycholeptics, indomethacin, mefenamic acid, niflumic acid, tenoxicam, morniflumate, tiaprofenic acid, oxaprozin, naproxen and esomeprazole, amtolmetine guacil, proglumetacin, cinnoxicam.
(TIF)

S3 Fig Prevalence of elderly opioid users, stratified by calendar year and individual drug. Combinations of oxycodone: oxycodone and naloxone, oxycodone and acetaminophen.
(TIF)

S4 Fig Yearly purchasing trend of non-opioid analgesics. Other NSAIDs: Dexketoprofen, mefenamic acid, niflumic acid, tiaprofenic acid, dexibuprofen, diclofenac combination, flurbiprofen, indomethacin, ketoprofen combination, ketorolac, lornoxicam, meloxicam, nabumetone, oxaprozin, tenoxicam.
(TIF)
