# Supplementary material for: Analgesic drug use in elderly persons: A population-based study in Southern Italy
Source: PLoS One. 2019 Sep 19;14(9):e0222836. doi: 10.1371/journal.pone.0222836 (PMC6752879; doi:10.1371/journal.pone.0222836)
Supplement: S1 Table — (PDF) [file pone.0222836.s001.pdf]

Analgesic drug use in elderly persons: a population-based study in Southern Italy

Ylenia Ingrasciotta[1*], Janet Sultana[1*], Francesco Giorgianni[2], Enrica Menditto[4], Angelo Scuteri[5], Michele Tari[6], Daniele Ugo Tari[6], Giorgio Basile[1], Gianluca Trifiro'[1,3**]

[1] Department of Biomedical and Dental Sciences and Morphofunctional Imaging, University of Messina, Messina, Italy

[2] Unit of Clinical Pharmacology A.O.U. ''G. Martino' Hospital', Messina, Italy

[3] Department of Medical Informatics, Erasmus Medical Center, Rotterdam, the Netherlands

[4] CIRFF, Center of Pharmacoeconomics, University of Naples Federico II, Naples, Italy

[5] HSR Pisana IRCCS, Rome, Italy

[6] Local Health Unit of Caserta, Caserta, Italy

*Joint first authors

**Corresponding author email: trifirog@unime.it; telephone: +39 090 2213647; fax: +39 090 2213300

S1 Table: Analgesics identified by ATC codes and generic name.

Category	ATC	Generic name
Non-steroidal anti-inflammatory drugs	M01AB01	Indometacin
	M01AB05	Diclofenac
	M01AB14	Proglumetacin
	M01AB15	Ketorolac
	M01AB16	Aceclofenac
	M01AB55	Diclofenac, combinations
	M01AC01	Piroxicam
	M01AC02	Tenoxicam
	M01AC05	Lornoxicam
	M01AC06	Meloxicam
	M01AE01	Ibuprofen
	M01AE02	Naproxen
	M01AE03	Ketoprofen
	M01AE09	Flurbiprofen
	M01AE11	Tiaprofenic acid
	M01AE12	Oxaprozin
	M01AE14	Dexibuprofen
	M01AE17	Dexketoprofen
	M01AE52	Naproxen and esomeprazole
	M01AE53	Ketoprofen, combinations
	M01AE91	Carprofen
	M01AG01	Mefenamic acid
	M01AH01	Celecoxib
	M01AH05	Etoricoxib
	M01AX01	Nabumetone
	M01AX02	Niflumic acid
	M01AX05	Glucosamine
	M01AX17	Nimesulide
	M01AX22	Morniflumate
	N02BA01	Acetylsalicylic acid
	N02BA51	Acetylsalicylic acid, combinations excl. psycholeptics
Weak opioids	N02AA59	Codeine
	N02AX02	Tramadol
Strong opioids	N02AA01	Morphine
	N02AA03	Hydromorphone
	N02AA05	Oxycodone
	N02AA55	Oxycodone and naloxone
	N02AB03	Fentanyl
	N02AE01	Buprenorphin
	N02AX06	Tapentadolo
	N07BC02	Methadone
