# Supplementary figures and images for: Analgesic drug use in elderly persons: A population-based study in Southern Italy
Source: PLoS One. 2019 Sep 19;14(9):e0222836. doi: 10.1371/journal.pone.0222836 (PMC6752879; doi:10.1371/journal.pone.0222836)

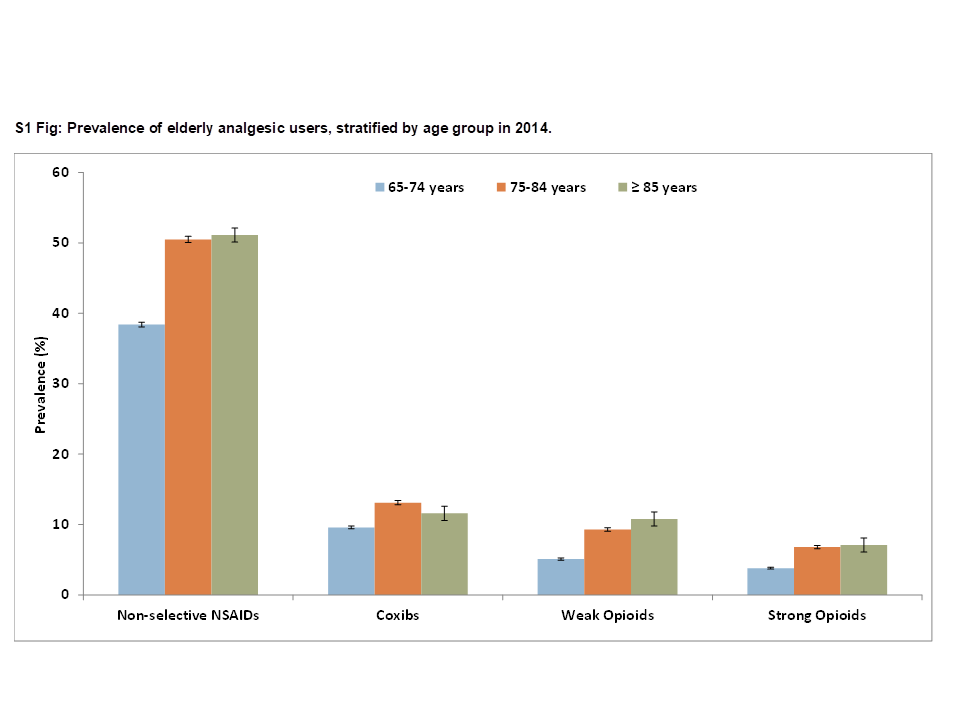

Supplement: S1 Fig — (TIF) [file pone.0222836.s002.tif]

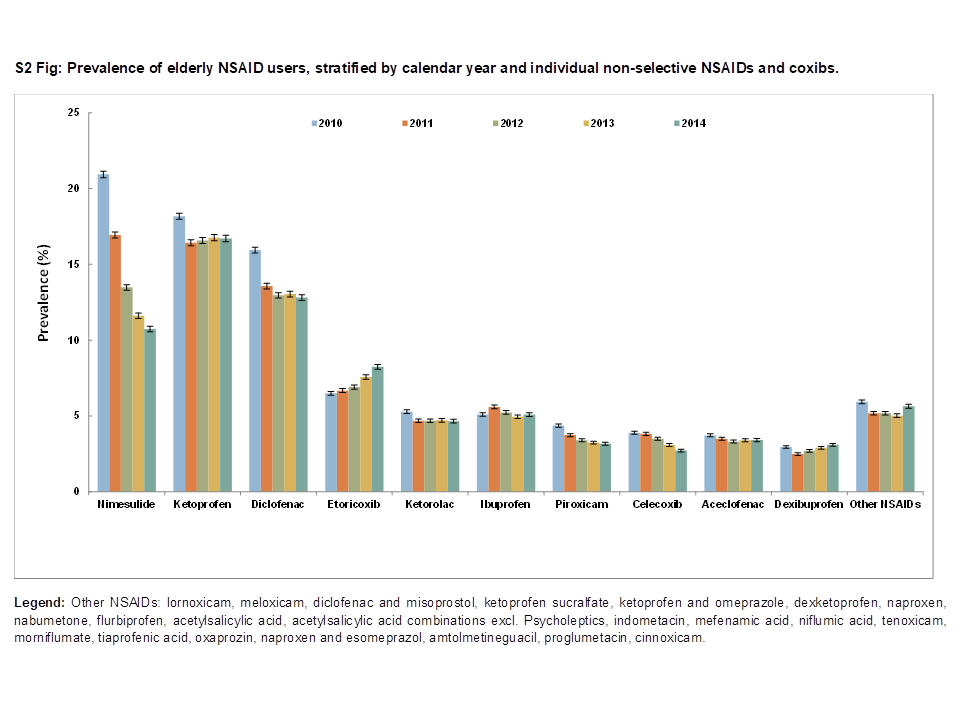

Supplement: S2 Fig — Other NSAIDs: lornoxicam, meloxicam, diclofenac and misoprostol, ketoprofen sucralfate, ketoprofen and omeprazole, dexketoprofen, naproxen, nabumetone, flurbiprofen, acetylsalicylic acid, acetylsalicylic acid combinations excl. Psycholeptics, indomethacin, mefenamic acid, niflumic acid, tenoxicam, morniflumate, tiaprofenic acid, oxaprozin, naproxen and esomeprazole, amtolmetine guacil, proglumetacin, cinnoxicam. (TIF) [file pone.0222836.s003.tif]

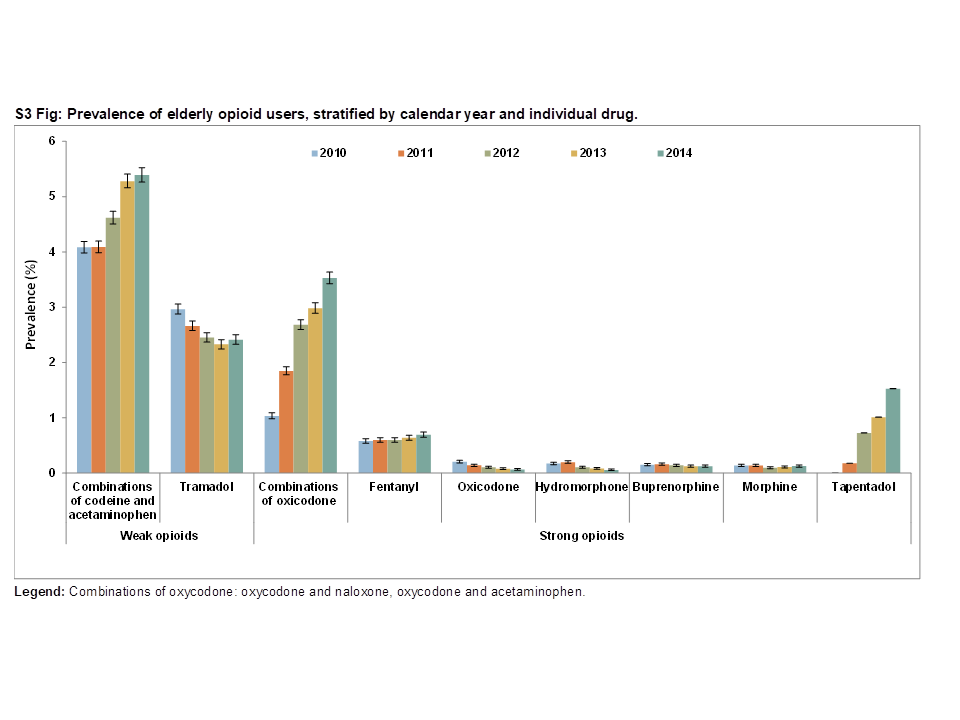

Supplement: S3 Fig — Combinations of oxycodone: oxycodone and naloxone, oxycodone and acetaminophen. (TIF) [file pone.0222836.s004.tif]

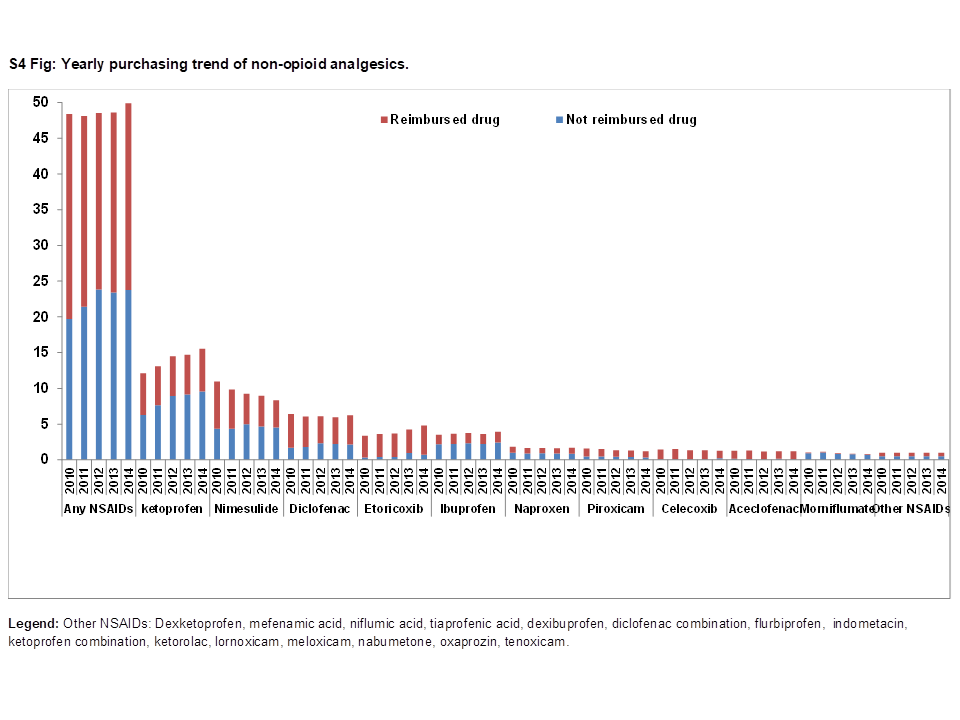

Supplement: S4 Fig — Other NSAIDs: Dexketoprofen, mefenamic acid, niflumic acid, tiaprofenic acid, dexibuprofen, diclofenac combination, flurbiprofen, indomethacin, ketoprofen combination, ketorolac, lornoxicam, meloxicam, nabumetone, oxaprozin, tenoxicam. (TIF) [file pone.0222836.s005.tif]
